# Patterns and biases in climate change research on amphibians and reptiles: a systematic review

**DOI:** 10.1098/rsos.160158

**Published:** 2016-09-07

**Authors:** Maiken Winter, Wolfgang Fiedler, Wesley M. Hochachka, Arnulf Koehncke, Shai Meiri, Ignacio De la Riva

**Affiliations:** 1 WissenLeben e.V., Raisting, Germany; 2 Max Planck Institute for Ornithology, Radolfzell, Germany; 3 University of Konstanz, Konstanz, Germany; 4 Lab of Ornithology, Cornell University, Ithaca, NY, USA; 5 WWF Germany, Berlin, Germany; 6 Department of Zoology, Faculty of Life Sciences, Tel Aviv University, Tel Aviv, Israel; 7 Museo Nacional de Ciencias Naturales-CSIC, Madrid, Spain

**Keywords:** amphibia, climate change, bias, Linnean shortfall, reptilia, Wallacean shortfall

## Abstract

Climate change probably has severe impacts on animal populations, but demonstrating a causal link can be difficult because of potential influences by additional factors. Assessing global impacts of climate change effects may also be hampered by narrow taxonomic and geographical research foci. We review studies on the effects of climate change on populations of amphibians and reptiles to assess climate change effects and potential biases associated with the body of work that has been conducted within the last decade. We use data from 104 studies regarding the effect of climate on 313 species, from 464 species–study combinations. Climate change effects were reported in 65% of studies. Climate change was identified as causing population declines or range restrictions in half of the cases. The probability of identifying an effect of climate change varied among regions, taxa and research methods. Climatic effects were equally prevalent in studies exclusively investigating climate factors (more than 50% of studies) and in studies including additional factors, thus bolstering confidence in the results of studies exclusively examining effects of climate change. Our analyses reveal biases with respect to geography, taxonomy and research question, making global conclusions impossible. Additional research should focus on under-represented regions, taxa and questions. Conservation and climate policy should consider the documented harm climate change causes reptiles and amphibians.

## 1. Background

Anthropogenic climate change will increase mean global temperatures by more than 4°C within this century if we do not reduce greenhouse gas emissions drastically and immediately [1]. Such changes will be likely to have devastating impacts on many species, because the intensity and speed of changes are unprecedented within the last millions of years [2]. A clear overview of the impacts of climate change on species is necessary for an informed discussion on the need for emissions reductions to minimize further effects of climate change on species, and on the need for conservation actions to enable species to cope with changing climates. However, demonstrating a direct impact of climate change on a species is extremely difficult because (i) species are influenced by a large array of abiotic and biotic factors [3,4]; (ii) species differ in their vulnerability to changing climatic conditions, depending on their adaptability, their exposure to climatic extremes and their sensitivity [5–7]; and (iii) climate change can affect species both directly (e.g. by causing heat stress or desiccation) and indirectly (e.g. by influencing disease outbreaks [8] or food availability [9]).

Within single studies on climate change effects, the picture is even more complex. Generally, studies demonstrate a correlation between a species' trait and a climatic variable. However, such findings cannot determine whether the relationship is causal [10]. Furthermore, it may not even be obvious if the detected relationship has a negative or positive effect on the species [11], especially if there is no yardstick against which to measure the observed changes [12]. Decreases in population size, range size and survival clearly have a negative impact on a species, but this is less clear for observations such as changes in phenology, body size and distribution shifts. The impacts of distribution shifts on species will depend on factors such as habitat connectivity, competition and the availability of habitat and food—which can also be influenced by climate change [13].

Despite the large amount of research being conducted on the effects of climate change on animals, many important questions remain without clear answers. Which climate change effects are most prevalent? Are those effects negative, neutral or positive for the species? Are the effects generalizable across regions and taxa? Are there biases in study design that might influence the conclusions? Are there taxa, regions and questions on which future research should focus? Systematic reviews—the combined analysis of the results of different studies—enable us to answer some of those questions by determining if results of single studies are consistent across regions and taxa [14].

We conducted a systematic review of published studies on amphibians and reptiles to look for evidence both of systematic biases in the types of research being conducted and of patterns of climate change effects. Ectothermic vertebrates are likely to be more directly impacted by climate change than other vertebrates, because their body temperature depends on the surrounding temperatures (but see [15]). Both groups are very sensitive to environmental change and are declining worldwide [16–22]. These declines have been partly attributed to climate change [21,23,24]. Most amphibians have highly permeable skin, and both aquatic and terrestrial life stages. These attributes make them very sensitive to changes in temperature and precipitation. In contrast, reptiles are known to favour warm areas and might therefore be less severely affected by increasing temperatures or may even benefit from global warming.

To determine the identified effects of climate change on amphibians and reptiles, we conducted a systematic review on 104 original research papers. This analysis clearly indicated that both amphibians and reptiles are affected by climate change, but the rates at which effects of climate change are reported varied among geographical regions, species groups and research methods.

## 2. Methods

### 2.1. Literature search

We searched the Web of Science on the portal available through the Potsdam Institute for Climate Impact Research (PIK) for articles that investigated current or future effects of various climatic factors on amphibians and reptiles. The initial criteria for article selection can be seen from the search terms listed in the electronic supplementary material, table S1. This initial search gave us a list of 1818 articles. We also included studies (*n* = 123) that we found in the process of contacting authors and reading other articles. After initial screening, we still had to assess 539 articles in more detail. Only 104 of those articles fit our selection criteria and were included in the analysis. The selection criteria were

(1) Articles had to be published between 2005 and April 2015, because we wanted to focus on the most recently published studies. Studies that were conducted before 2005 have already been included in previous reviews [23,25,26].
(2) The study included climatic factors, i.e. any factor that is directly affected by climate change. This includes, for example, temperature, precipitation, number of dry days, water temperature and storms (for a full list of climatic variables, see electronic supplementary material, table S2).
(3) The study was based on data collected in the field and conditions were not experimentally altered.
(4) Data were collected over a period of at least 5 years [27,28], including studies whose data (i.e. distributional information) came from museum specimens. We also included studies whose data were collected in disjunctive time intervals that were at least 5 years apart, and studies that modelled future distributions based on current distribution data from atlas data or long-term studies.
(5) The study analysed observed patterns to determine current or future effects of climate change, including
  (a) a potential change of a trait (e.g. breeding date, distribution or body size) over time;
  (b) a potential correlation between the temporal change of a species’ trait with changes in some facet of climate (e.g. temperature and rainfall volume);
  (c) a potential change of some facet of climate over the period of the study; and
  (d) a before–after comparison that documents the impacts of severe weather events.
(6) The original studies had to report the statistics needed to run a systematic review (i.e. *p*-values that are needed to assess whether associations with climate were unlikely to have been observed by chance). See below for explanation for using *p*-values and not more detailed information such as effect sizes.

Studies that reported a statistically significant relationship between a climate variable and a trait (*p* < 0.05) were categorized as showing a climate change effect and those with no statistically significant relationships (*p* > 0.05) were categorized as not showing a climate impact. Ideally, we would have considered the statistical power of the reviewed studies to detect significant relationships. However, this was not possible, because few studies included such estimations of statistical power. We used *p*-values rather than the sizes of reported effects (i.e. statistical effect sizes) because of the diversity of responses in the reviewed studies (see electronic supplementary material, table S2). Lack of consistency in the types of analyses and results reported further made the use of effect sizes impossible. Preliminary attempts to obtain more detailed statistical data directly from authors were mostly unsuccessful and were therefore not extended to all authors. Thus, our analyses were constrained by the limitations of the available data to using ‘vote counting’ rather than using formal meta-analytic methods [29].

### 2.2. Data extraction

From each of the reviewed studies, we extracted information on the location (latitude, longitude and elevation) of the study site, the investigated species, the predictor (e.g. climatic data) and response variables (such as population size), and the results (see electronic supplementary material, table S2). Studies were assigned to one of the continents. North America was defined as Canada, the USA and Mexico. Continents and biogeographic realms were essentially identical as geographical units: only one species in Mexico fell into a different set depending on whether continents or biogeographic areas were used to group species. We made the pragmatic decision to group species by continent, because management actions are often based on political and not biogeographic regions. For large-scale studies, we estimated the central location among all study sites and determined latitude and longitude for this central point. Global studies received missing values for the location information. We determined altitude for those studies that gave the exact geographical location. However, that left us with a dataset with many unknown elevation data. Therefore, elevation was not included in the analysis. Species' data included information on taxonomy and conservation status. We standardized all species names to the taxonomic databases of amphibians (AmphibiaWeb [30]) and reptiles (The Reptile Database [31]).

In our description of the papers that we reviewed, we categorized the responses examined by researchers into 24 possible types, some of which were rarely investigated; the numbers of studies testing for any given class of response ranged from 1 to 27. The only classes of responses represented in 20 or more studies were: change in phenology (*n* = 20), change in population size (*n* = 21), change in occurrence probability (*n* = 25) and change in distribution (*n* = 27). There were insufficient studies examining each of the response classes to allow an examination of patterns within each of the classes of responses separately; we therefore collapsed information from the multiple types of responses into two variables that we used as responses in our analyses: a binary variable (‘SigClimateEffect’ in the archived data) describing whether a statistically significant response to climate change was identified, and a three-category variable (‘CCEffectOnSpecies’) identifying whether effects were positive (1), negative (−1) or not statistically detected (0). Some studies (32 of 109) examined more than one response variable, with the numbers of responses examined ranging from 1 to 4 (*n* = 77, 21, 10 and 1 studies with each number of responses, respectively). For studies examining multiple responses, we still condensed the results into a single value of SigClimateEffect and CCEffectOnSpecies. SigClimateEffect was given a value of ‘1’ if any one of the response variables was found to respond to climate change. CCEffectOnSpecies was similarly coded, except for the case in which responses in different directions were reported; in that case we set CCEffectOnSpecies to be a missing value. While this coding enabled us to combine data from studies with a diversity of response variables, any conclusions that we draw are potentially conditional on the mixture of response types that the authors of the reviewed papers chose to study.

Because of the large variety of predictor variables examined in the reviewed studies, our analyses were based on three broad functional groupings of predictors: (i) climatic variables as described above; (ii) human impact variables (such as habitat destruction, fragmentation, invasive species and pollution); and (iii) other environmental characteristics (such as disease, radiation and vegetation cover). The responses examined by the studies were similarly grouped into functional classes: (i) population size; (ii) distribution; (iii) phenology; (iv) morphology; (v) presence of disease (i.e. Chytridiomycosis); (vi) physiology; and (vii) genetic traits. The electronic supplementary material, table S2 lists the original predictor and response variables, as well as the way in which these were grouped into broader functional categories for analyses.

We determined the threat status for each species from the IUCN Red List (http://www.iucnredlist.org, accessed 20 May 2015), and combined the status categories into the two groups: ‘not threatened’ (least concern and near threatened) and ‘threatened’ (vulnerable, endangered, critically endangered). Data from species that were categorized as ‘data deficient’ or for which no assessment existed at the time of analysis were removed from all analyses that examined threat status.

### 2.3. Statistical analysis and interpretation

All data were analysed using R [32]. Because of the binary nature of the response variables (electronic supplementary material, table S2), all analyses were done using logistic regressions with the ‘glm’ function within the core ‘stats’ library of R. Probabilities of effects in logistic regressions were calculated using likelihood-ratio tests in the ‘anova.glm’ function associated with ‘glm’. The tests of independence were done using the ‘chisq.test’ function in R's ‘stats’ library, and the chi-squared goodness-of-fit probabilities were calculated manually. The specific predictor variables and forms of all statistical models are described in the Results section. Several species were investigated in more than one study; when this occurred, each species in each separate study was treated as an independent data point. For this reason, sample size in most analyses is higher than the number of species investigated by all studies combined (*n* = 313 species and 464 species–study combinations). About 36% (37 of 104) of the studies that we reviewed reported results for multiple species. Again, for analyses in which species was the unit of response, we treated each species within each study as a separate data point. For other analyses, each study was an independent data point (*n* = 104), and in these cases all studies were treated equally, regardless of the number of species that were investigated by the studies.

We are aware that our approach might bias the results if findings for multiple species within a study were not independent. For example, study methodologies might have widely varying probabilities of detecting true effects of climate change. There are also biologically real reasons for non-independence including: shared geographical location, phylogenetic similarity and same study methods being applied across all of the species investigated. We explicitly tested for the presence of some of these possible biases—geographical differences among broad regions, differences in the response variables examined, taxonomic effects at the level of class and family. However, the quantity and nature of the available data made it impossible to use elegant statistical solutions. Specifically, we could not treat ‘study’ as a statistical random effect, because a large proportion (64%; 67 of 104) of studies reported results only from a single species. In this circumstance, random effects of study cannot be estimated, and mixed models failed to converge to solutions in all of our preliminary trials. We also attempted to control for non-independence with analyses in which the response variable was an odds ratio (i.e. each response was two numbers: the number of species with a response and the number with no response). While in some cases the use of a study-wide odds ratio appeared to function as desired, in most cases, the results—regression coefficients and their standard errors and *p*-values—were identical, regardless of whether each species–study combination or each study was used as a single datum in our analyses. In effect, a *de facto* conclusion of our study is that future research needs to quantify the manner in which study methodology determines whether a biologically real effect of climate change is identified. Within the context of this study, our conclusions need to be viewed as the most accurate assessment of the existing climate change research on amphibians and reptiles given the diverse and uncoordinated nature of the types of studies conducted within the last decade.

The use of vote counting analyses rather than use of formal meta-analyses can be problematic (see chapter 28 of [33]). Specifically, ‘no’ votes (i.e. *p*-values > 0.05) can result either if there is no biologically real effect or if statistical power is insufficient to detect a biologically real effect. Analyses that failed to detect an effect thus need to be interpreted in the light of this fact: failure to detect specific effects of climate change means that such effects either truly do not exist, or were undetected because the effects were too subtle to be statistically detected given the typical effort expended in the studies that we have reviewed. Thus, if anything, our review is under-reporting rates with which climate change has affected species.

## 3. Results

Our literature search identified 539 articles that met the initial criteria for further examination, 288 on amphibians and 251 on reptiles. Of those studies, only 104 fit our specific selection criteria, 62 on amphibians and 42 on reptiles (hereafter termed ‘reviewed studies’; see electronic supplementary material, table S3). The reviewed studies investigated 313 species, 195 amphibians and 118 reptiles; eight taxa were not identified to species level, but were still included in our analyses as taxonomic units analogous to species (electronic supplementary material, table S4). The species investigated in the reviewed studies represent 2.7% and 1.2% of all described amphibian and reptilian species, based on our taxonomic sources (7416 amphibian species in http://amphibiaweb.org/amphib_names.txt, accessed on May 26 2015; and 10 178 reptilian species listed in http://www.reptile-database.org/data/, release 23, March 2015).

### 3.1. Biases

We first investigated five potential biases, because failure to identify biases can influence the interpretation of the results from this review: (i) geographical bias of the locations of the reviewed studies (Wallacean shortfall); (ii) uneven taxonomic representation of the species investigated (Linnean shortfall); (iii) uneven representation of threatened species within the data; (iv) failure to consider alternative explanations for observed responses; and (v) uneven representation of potential responses examined among the studies.

#### 3.1.1. Geographical bias

**Figure 1.**
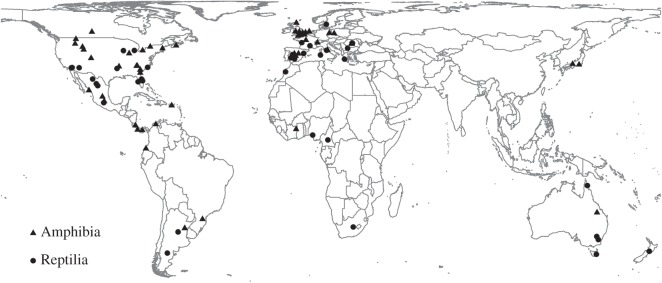
Locations of the reviewed studies on amphibians (triangles) and reptiles (circles). Three global-scale studies were not included in this map.

While the reviewed studies were based on data collected throughout the world, there were strong differences in the proportions of studies conducted among continents. Seventy per cent of all studies were conducted in Europe and North America, both for amphibians and reptiles (figure 1 and electronic supplementary material, table S5). The other continents or regions, most of which have much higher species diversity, were covered much less thoroughly (for example, no studies that fit our selection criteria were conducted anywhere between longitudes 26° and 136° east).

#### 3.1.2. Taxonomic bias

**Figure 2.**
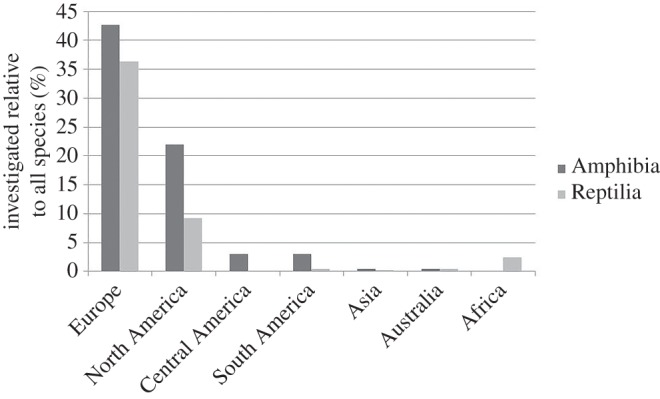
Percentage of species investigated by the reviewed studies within a continent, relative to all species within it. For details, see the electronic supplementary material, table S5.

Owing to this geographical bias, a large percentage of all known European and North American species were investigated, whereas species from other continents were severely under-represented (figures 1 and 2; electronic supplementary material, table S5). For example, in Europe 43% of all amphibian and 37% of all reptilian species were represented in at least one study. At the same time, Europe harbours only 1.2% and 1.5% of all described amphibian and reptilian species of the world. In contrast, only 1.0% and 0.06% of all South American amphibian and reptilian species were included in the reviewed studies. Together, the species investigated by the reviewed studies belonged to only 32% and 26% of all amphibian and reptilian families.

The amphibians investigated belonged to 24 of the 74 amphibian families, representing only 2.7% of all described amphibian species (table 1 and electronic supplementary material, table S6). Even the relatively well-known order of frogs and toads, the Anura, was represented by only 31% of its families (table 1). The most species-rich amphibian family, the Hylidae, was represented by only 24 of 950 species. Other species-rich families, such as Dendrobatidae with 303 known species and Hyperoliidae with 223 known species, were not represented at all (electronic supplementary material, table S6).

**Table 1.** Comparison of the number of amphibian and reptilian families per order (‘families per order’) with the number of families studied by the reviewed literature (‘families studied’). A complete list of all families and species in the reviewed studies is provided in the electronic supplementary material, tables S4 and S6.

class	order	families per order	families studied	% families studied per order
Amphibia	Anura	54	17	31
	Caudata	10	5	50
	Gymnophiona	10	2	20
Reptilia	Crocodilia	3	0	0
	Rhynchocephalia	1	1	100
	Squamata	66	16	24
	Testudinata	18	5	28

For reptiles, 22 of the 88 families were included in the reviewed studies, representing 1.5% of all described reptilian species (table 1 and electronic supplementary material, table S6). The most species-rich family, the Scincidae (skinks) with 1589 species, was represented by only 11 species. Other species-rich families such as the Agamidae and Dactyloidae (anoles) were not investigated by any of the reviewed studies (electronic supplementary material, table S6).

While the species that have been studied do not provide an even representation of the world's families of amphibians and reptiles, the families that were studied are proportionally representative of the seven orders of amphibians and reptiles. The proportions of families per order did not differ from that expected under equal probability of study (*χ*^2^ contingency table test: *χ*^2^ = 7.17, d.f. = 6, *p* = 0.31, *n* = 464).

#### 3.1.3. Bias in the conservation status of the species investigated

Of the 313 species investigated by the reviewed studies, 258 had been assessed by the IUCN Red List and were not ‘data deficient’. Seventeen per cent of those assessed species were categorized as ‘threatened’—15% of the assessed amphibians (26 out of 173 species) and 22% of the assessed reptilians (19 out of 85 species). Considering amphibians (41% classified as threatened; http://www.iucnredlist.org) and reptiles (19% threatened) together, there was a disparity between the observed and expected proportions of threatened species that were studied (*χ*^2^ = 28.96, d.f. = 1, *p* < 0.001). This statistical significance was largely the result of the small proportion of threatened amphibian species that were studied.

Given that an effect of climate change was reported for a species in a study, a species' status (i.e. threatened or not threatened) had no detectable association with the reporting of an effect of climate change. This conclusion applied to both positive and negative effects of climate change, and no differences were found between amphibians in the effect of threat status (table 2).

**Table 2.** Association between IUCN threat status of species and the probability that a study will suggest a negative or positive impact of climate change on the species (‘prob climate effect’). Presented are estimated probabilities of identifying negative or positive impacts of climate change in the reviewed studies depending on the class and the species’ population status. Some species are represented more than once because each combination of species and study was treated as a separate data point (*n* = 390); species without population status information and ‘data deficient’ species were excluded from the analysis. Data were fit with a logistic regression in which the model predicted the probability that a study detected/predicted a negative or positive effect of climate change. Categorical predictor variables were the taxonomic class of the species, whether its threat status was ‘threatened’ or ‘not threatened’, and the interaction between these two potential effects. Probabilities and 95% confidence limits are presented.

class	status	prob climate effect	lower 95	upper 95	*p* (class)	*p* (status)	*p* (class × status)
probability of finding a *negative* impact of climate change on the species							
Amphibia	not threatened	0.56	0.50	0.62	0.17	0.94	0.99
Amphibia	threatened	0.57	0.38	0.73			
Reptilia	not threatened	0.65	0.51	0.77			
Reptilia	threatened	0.65	0.44	0.82			
probability of finding a *positive* impact of climate change on the species							
Amphibia	not threatened	0.04	0.02	0.07	0.0005	0.95	0.81
Amphibia	threatened	0.03	0.004	0.21			
Reptilia	not threatened	0.16	0.08	0.29			
Reptilia	threatened	0.17	0.07	0.39			

#### 3.1.4. Bias in the predictor variables used when studying climatic effects

Studies on the effects of climate change which only investigate climatic variables are assumed to be biased towards finding a climatic effect, because such studies neglect the fact that species’ attributes can also be influenced by other factors. These other factors include, for example, human impact factors such as habitat destruction and pollution, or changes in environmental factors such as vegetation cover, radiation and presence of disease (for a detailed list of all variables considered, see electronic supplementary material, table S2). More than half of the reviewed studies did not investigate alternative hypotheses. Only 14 out of the 104 studies investigated variables from each of the three main groups of independent variables (table 3).

**Table 3.** Number of studies that include at least one of the three main types of factors that can affect the investigated species, and probability that this factor-combination was investigated by the reviewed studies.

main factors investigated^a^	prob. of investigation	*n*
climatic effects only	0.57	59
climatic and environmental effects	0.23	24
climatic and human impact effects	0.07	7
climatic, environmental and human impact effects	0.13	14

^a^Main factors include: climatic factors (such as temperature and precipitation), environmental factors (such as vegetation cover and competition), and direct human impacts (such as habitat destruction and fragmentation). For a complete list of variables see electronic supplementary material table S2.

Perhaps surprisingly, the proportion of species for which an effect of a climatic variable was reported did not differ between studies that only investigated climatic factors (proportion = 0.81, 95% CL: 0.73–0.84) and those that included more than one factor in their model (proportion = 0.81, 95% CL: 0.75–0.86). This difference was not statistically significant (*p* = 0.55, *n* = 432 species–study combinations, *n* = 104 studies). The listed *p*-values are the probabilities from logistic regressions testing for differences in the rates of detection of climate effects dependent on whether only climate predictors were considered in the study or not.

#### 3.1.5. Bias in the investigated response

The reviewed studies investigated a large number of potential species’ responses to climate change (electronic supplementary material, table S2). These types of responses studied were not equally represented in the reviewed studies even when collapsing the initial 19 classes of responses into nine functional groups: the most commonly investigated climatic effects on species were changes in population size, changes in distribution and changes in phenology or survival. Few long-term studies described potential climatic effects on reproduction, disease prevalence, morphology or physiology (table 4).

**Table 4.** Percentage of studies investigating a certain type of species’ response.

response type investigated^a^	percentage of studies	*n*^b^
change in population size	29	41
change in distribution	19	28
change in timing	14	20
change in survival	14	21
change in reproduction	9	14
change in morphology	7	10
extinction probability	5	7
change in disease prevalence	3	4
physiological changes	1	2

^a^For a detailed description of the variables included in the different response categories, see electronic supplementary material table S2.

^b^*n* is higher than the total number of studies, because several studies investigated more than one response type.

Across all types of responses investigated, studies for which the authors collected data in the field were more prevalent than studies that used data from the literature or other databases (60 field versus 44 modelling studies). The numbers of species examined in a study also typically did not vary between field and modelling studies: in both cases, the median number of species per study was 1; 75% quartiles were 3.5 and 3 species for field and modelling studies, respectively. However, studies of change in distribution were almost entirely based on forecasting future distributions from climate envelope models, 22 of 27 studies on distribution change. Changes in the distribution of a species in response to climate change were far more likely to be reported in studies for which climate envelope modelling was used than in studies that obtained data from field observations (*p* = 0.0002, *n* = 180; logistic regression): the predicted probability of detecting changes in distribution was 47% for field studies (95% CI: 24–71% when each species was treated as an independent datum) versus 88% (82–92%) for studies that used climate envelope models.

Our data indicate that forecasting possible future impacts of climate change is qualitatively different than studying the impacts of climate change that have happened to date based on field data. In addition, forecasting studies were associated with a far higher probability of reporting impacts of climate change. Because of this, and because climate projection studies were the minority of the studies reviewed, we have excluded all data from the climate projection models in the analyses that follow.

### 3.2. Directions of the effects of climatic variables

Even after we excluded all studies that forecasted potential future impacts of climate change, we found that a large proportion of studies reported effects of climate change on species. In Europe 20 out of 21 amphibian and four out of five reptilian species were affected by climate change, as well as 26 out of 48 amphibian species and four out of five reptile species in North America. Of the impacted species, 62% of amphibians (56 of 90 species) and 55% of reptiles (six of 11 species) were reported to have been negatively affected, mainly through population declines, reductions in habitat suitability and reduced survival and range sizes. We did not use data from phenological studies in these analyses because they do not directly affect populations.

#### 3.2.1. Geographical and taxonomic differences in presence and direction of effects

Geographical bias in the distribution of studies resulted in low sample sizes for several continents, as described above. Thus, we had to restrict the analysis on data from Europe and the Americas to statistically investigate if the probability of an effect of climatic variables on amphibians and reptiles varied among geographical areas. There were insufficient data to estimate probabilities of reporting effects of climate change within South America. For the other regions, the estimated probabilities of reporting climate change effects were over 50%, although the probabilities differed among continents (*p* > 0.0001; table 5). These differences did not vary among classes as neither the taxonomic class (*p* = 0.86) nor the continent × class interaction (*p* = 0.78) were statistically significant. In Europe, about 90% of amphibian and reptilian species were reported to have been affected by climatic variables. In contrast, studies within North and Central America only reported climate change effects in roughly 50% and 65% of the species, respectively (table 5).

**Table 5.** Probabilities of detecting effects of climatic variables, and their variation among continents and between classes of amphibians and reptiles. The probabilities and their 95% confidence limits were calculated from a logistic regression in which continent, class and a continent × class interaction were the predictors. The response was binary with ‘1’ meaning that an effect of climate on some aspect of the species’ biology was reported. Each species within each study was treated as an independent data point (*n* = 412). Only data from Europe, North America, Central America, and South America were used because of small sample sizes for other geographical areas. Even within the set of regions with larger sample sizes, insufficient data were available to estimate probabilities of reporting effects of climate change for either amphibians or reptiles in South America.

Continent	class	prob	lower_95_	upper_95_
Europe	Amphibia	0.90	0.77	0.96
Europe	Reptilia	0.89	0.49	0.99
North America	Amphibia	0.52	0.41	0.61
North America	Reptilia	0.57	0.22	0.86
Central America	Amphibia	0.62	0.42	0.78
Central America	Reptilia	0.67	0.25	0.92

**Figure 3.**
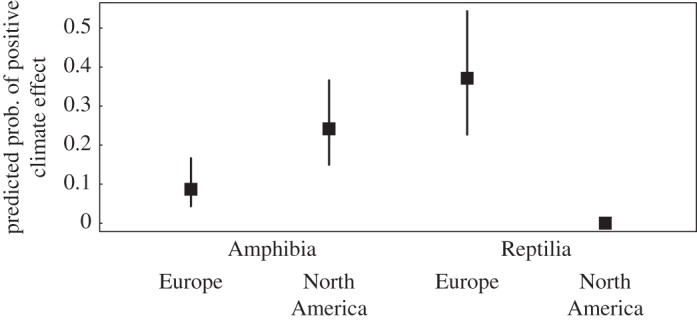
Variation between Europe and North America, and between classes, in whether effects of climatic variables were assessed as being positive for the species investigated. We included only those species for which the reviewed studies reported a positive or a negative response, excluding those with no or variable responses (*n* = 210). The figure presents results based on the species–study data points, because the results were very similar to the analyses on studies only.

The above analyses combined negative and positive effects of climate change, but there might be difference in the rates at which positive and negative effects were reported. Thus, we also estimated the probabilities of reporting positive effects of climate change on amphibians and reptiles relative to the probabilities of reporting negative effects. Sample sizes for these analyses were smaller than for the previous analyses, and only data from North America and Europe were used. We found that the probability of demonstrating a positive effect of climate on species differed between regions and between taxonomic classes. The pattern reversed between North America and Europe, leading to a class × continent interaction that was significant (*p* = 0.01). The predicted values illustrate this reversal (figure 3): in North America the probability of finding a positive effect of a climatic variable on amphibians was greater than in Europe, whereas in Europe there was a slightly higher likelihood that studies found a positive response from reptiles. In fact, no single study of reptiles in North America predicted a positive outcome for reptiles in response to climate change, and hence no estimate of statistical uncertainty could be calculated. The likelihood that a reviewed study described a negative effect of a climatic variable on a species is the opposite to the likelihood of showing a positive effect, so no results are presented for this view of the data.

#### 3.2.2. Taxonomic patterns

The final question that we considered was whether there were differences across families in the rates at which effects of climate change were reported. We limited these analyses to a subset of the data that included only those families for which at least five species were represented, in order to allow for the potential of some variation in response within the families examined. We further restricted our data to studies that looked for effects of climate change on only a single class of responses, changes in population size or occurrence rates, to remove potential biases that could result from differences in the rates at which effects of climate change were reported for studies of different types of responses. Only four additional studies of amphibians would have been added if we had considered all types of responses together. Reports of positive and negative responses to climate change were examined in separate analyses.

On average across all families of amphibians examined, the probability of finding a negative effect of climatic variables on population size was much higher than the probability of finding a positive effect (table 6). We found a statistically significant difference among families in the probabilities of reporting negative effects of climate change. However, probabilities of reporting positive effects of climate change did not vary significantly across families.

**Table 6.** Phylogenetic consistency in the probability of detecting negative and positive effects of climate change on the distributions of amphibians. This table presents the expected probabilities (with 95% confidence intervals) that the reviewed studies of change in population size reported significant negative or positive effects of climate change for species within each family that was represented by five or more data points. These predicted values come from a logistic regression in which the predictor variables were taxonomic family. Only amphibians and not reptiles were represented with sufficient studies to conduct this analysis. The response variables were binary, recording either whether a study reported a negative effect of climate change (versus no identifiable effect or a positive effect) or whether a study reported a positive effect of climate change. While the data (*n* = 163) contain some instances in which individual species are represented by multiple data points, the vast majority of the data points represent unique species (compare the ‘*n*’ and ‘no. spp’. columns that present counts of the numbers of data points and the number of species represented by these data). When all studies for a family reported the same conclusion it was not possible to estimate confidence limits around predictions and the probability of a study reporting a negative or positive effect is either 1 (all studies reporting that type of effect) or 0 (none of the studies show that type of effect).

			probability of showing a *negative* effect				probability of showing a *positive* effect			
amphibian family	*n*	no. spp.	prob. negative	lower 95% limit	upper 95% limit	*p* (family)	prob. positive	lower 95% limit	upper 95% limit	*p* (family)
Ambystomatidae	6	4	0.83	0.36	0.98	0.03	0.17	0.02	0.64	0.24
Bufonidae	11	9	0.18	0.04	0.51		0.18	0.04	0.51	
Craugastoridae	6	6	0.83	0.36	0.98		0.00			
Eleutherodactylidae	5	5	0.80	0.30	0.97		0.00			
Hylidae	7	7	0.75	0.22	0.86		0.29	0.07	0.68	
Ranidae	8	7	0.75	0.37	0.94		0.00			

## 4. Discussion

### 4.1. Reported effects of climate change

The reviewed studies reported a high prevalence of climate change effects on amphibian and reptilian populations; the majority of these effects had negative consequences for amphibian and reptile populations. This much would be expected based on prior reviews on climate change effects on species generally [22,33] and specifically on amphibians [13,16,34] and reptiles [35–38]. In this paper, we have gone further than a basic summary of findings, having explored the geographical and taxonomic generality of conclusions that can be drawn from the studies that have been conducted within the last decade.

The prevalence of studies that indicate climate effects could be caused by a publication bias towards significant results ([39,40], but see [41]). However, we found that effects of climate change were reported with similar probabilities regardless of whether climate change was the only predictor examined in a study or whether multiple alternative causes of change were examined. We suggest that this finding is an encouraging indication of minimal reporting bias even in studies whose sole intent was to examine climate change as a factor impacting amphibian or reptile populations.

Our results indicate that climate change can be a significant cause for the current population declines in amphibian and reptilian species [18,42,43], and are therefore consistent with studies that concluded that climate change is an important factor affecting many species [23,44], including amphibians [13,16,33] and reptiles [34–37]. Two factors appear to contribute most to amphibian declines: destruction and fragmentation of habitat [45], and the fungal disease chytridiomycosis [46–49]. This disease has caused many extinctions of amphibian populations and species and is thought to be compounded by climate change [8,48–51]. However, the presence of a clear cause–effect relationship between climate change and chytridiomycosis is not universally accepted [52,53]. For reptiles, habitat loss, harvesting and invasive species on islands are described as main causes for declines [54]. It is debatable whether any single factor should be considered the biggest threat to amphibian and reptile species, as some authors note that single-factor causality is too simplistic [55–60].

We found that studies looking for future impacts of climate change had a much higher probability of reporting effects of climate change than studies looking for effects of climate change that have already occurred. Forecasting based on climate-envelope models indicated that future climate changes could cause serious declines in amphibian and reptile populations, mainly because habitat suitability will decrease [61–63], thermal tolerances might be exceeded [20,64] and—for species with temperature-dependent sex determination—increased sex bias might affect future reproductive output of populations [65,66].

Potentially, the high probability of reporting effects of climate change based on climate envelope models is misleading because these models are typically based on a simple space-for-time substitution [67]. Thus, the models assume that current-day spatial variation in climate *and* associated biotic and abiotic environmental features will provide a good indication of all aspects of the environments that species will face in the future when climatic zones have shifted. Climate envelope models can therefore only give a limited view on future effects of climate change if not all aspects of a species' biology, such as dispersal and survival, are taken into account [68,69]. Additionally, climate envelope modelling assumes an inability of animals to reduce the negative effect of increased temperatures by seeking out cooler microhabitats. One recent study [70] supports the assumption that behavioural adaptation will be minimal. However, for many species, the detailed information on ecology and life history needed to assess the assumptions of climate envelope models is missing [71].

Nevertheless, the conclusions from climate envelope models may be, in some cases, more reliable than conclusions based entirely on field studies. Current field studies have collected data from relatively short time periods (the average time span of the reviewed studies was 23 years), a time span over which climate change may not have been strong enough to cause a readily detectable effect. Based on the results of climate models, global mean temperature could increase by more than 4°C within this century. Modelling studies thus might even paint an optimistic picture if they assume that species only depend on abiotic variables, not taking into account a potential catastrophic disruption in the complex inter-relationships within ecosystems.

In spite of the prevalence of negative effects of climatic variables on amphibians and reptiles, it is important to note that climate change can also affect some species positively. Positive effects include range expansions in mountain areas [72] and into higher latitudes [73], increased winter survival of montane species [74] and faster development [75]. Care should be taken that studies and analyses are not biased towards the expectation that results will indicate a negative effect of climate change.

### 4.2. Biases in studies cause a neglect of important research topics

We found strong bias in geographical and taxonomic coverage of studies (Wallacean and Linnean shortfalls [76,77]) and in the questions that were studied. A bias towards northern regions might paint a less negative picture of climate change effects because species in cool areas, especially at the northern range limits, are more likely to benefit from warming. Another issue caused by this geographical bias is that high research intensity is in the areas of low species diversity. Geographical biases in research on amphibians and reptiles have previously been noted in other contexts, including reviews on amphibian ecotoxicology between 1969 and 2004 [78], on the importance of habitat change on amphibians and reptiles [79], on the status of reptiles of the world [54], on the robustness of global amphibian range maps [80] and on studies on biodiversity in general [81,82]. Each of these prior reviews recognized the problem of a strong bias towards studies being mainly conducted in North America and Europe.

The geographical bias is at least partially responsible for a taxonomic bias in which entire families of amphibians and reptiles, including families with hundreds of species, were not included in the studies we reviewed. Such taxonomic bias is a well-known problem [83]. Both the geographical and taxonomic bias might result in the investigated species disproportionally representing non-threatened species, because most threatened species occur in small ranges close to or south of the equator (IUCN Red list: http://www.iucnredlist.org/initiatives/amphibians/analysis/geographic-patterns), a potential bias that has already been noted in studies of marine mammals [84]. Nevertheless, we did not detect any effect of threat status on the likelihood of reported effects of climate change (table 2).

### 4.3. Extinctions

Extinctions could be the ultimate consequence of negative impacts of climatic variables [85]. However, even though amphibians are experiencing unprecedented declines and a large number of species and population extinctions have been documented during the last decades, climate change has rarely been identified as the main cause for any of the reported extinctions: Pounds *et al.* [8] attributed extinctions of Middle and South American amphibians to changing climatic conditions that favoured the chytrid fungus (*Batrachochytrium dendrobatidis*); and Sinervo *et al*. [20] related several extinctions of lizard populations to rising temperatures that surpassed the thermal tolerance of reptilian species. However, other studies found no evidence for chytridiomycosis being influenced by changing climates [52] or thermal tolerance being directly linked to extinctions in reptiles [86] or amphibians [87]. Most studies based on observations rather than on extrapolations from models cannot directly relate climate change to extinctions. In fact, all recently extinct reptiles, except the south African *Tetradactylus eastwoodae*, went extinct on islands, usually because of introduced predators or direct prosecution [88,89]. However, several models predict future species extinctions in both amphibians [55,90] and reptiles [20,64,91] due to climate change.

Even though studies have not shown that climate change directly affects species' extinction rates, it is likely that climate change will aggravate other stressors such as disease [92], habitat change and competition, thus increasing the likelihood of population declines and extinctions. This prediction is especially worrisome, because these stressors are most prevalent in the areas with highest species richness [55], and because extinction risks are concentrated in the areas of highest species diversity [93].

### 4.4. Recommendations for future research

There is a growing number of studies on the impacts of global climate change on species, including amphibians and reptiles [94]. This trend has greatly increased our knowledge of the current and potential future impacts of global climate change. Nevertheless, there are many regions, taxa and questions that are currently poorly represented by long-term studies on reptiles and amphibians. Information gaps exist for many regions of the world (especially in Africa, Asia and South America), on many threatened amphibian and reptilian taxa, and on several life-history traits such as reproduction and survival. Given the geographical and taxonomic differences in probabilities of responses to climate change that we found, we believe that it would be unwise to attempt to extrapolate conclusions from well-studied regions and taxa (figure 2).

In addition to the need for broader taxonomic and geographical representation, our review identified other weaknesses in the existing information that has been gathered. Mechanistic studies—studies focusing on questions on physiology, reproduction, juvenile recruitment and survival—are rare. In amphibians, it would be important to develop a clearer understanding of the survival probabilities across the entire life cycle, because a different set of factors will be likely to influence each stage [95]. The preponderance of climate envelope models with space-for-time substitutions [67] in the reviewed studies, and the need to test the assumptions of envelope modelling, is an important reason for encouraging a greater number of mechanistic studies.

The ways in which information is presented should be improved in order to increase the value of individual studies. In the process of collating data for our review, we found that many published studies do not report key information necessary to make them comparable to other studies, information such as effect sizes, power analyses or even just simple information such as the time spans of studies or the exact location(s) of study sites. These omissions of information forced us to restrict ourselves to analyses of presence or absence of reported patterns, whereas ideally we should have compared and contrasted quantitative differences in effects across studies.

Admittedly, the research needed to better anticipate the global impacts of climate change may not be conducive to rapid publication. The deficiencies in information can only be filled by conducting research in less convenient areas, sample sizes would be lower than for studies on common species in easily accessible areas, and resulting papers will be likely to get published in lower-ranking journals. Nevertheless, there are even gaps in our knowledge on common species within the best-studied regions of the world that can be targets of new research projects, such as distribution information for European amphibians and reptiles [96].

Finally, there is a great need for long-term studies in global change research, because species’ responses are often not detectable over short time periods [97–99] and because species’ responses can lag behind observed environmental changes [100]. While for career and funding reasons studies over only a few years are forming the vast majority in today's research, these studies are of limited value for the detection of biologically significant long-term trends in distribution, population size or reproduction. Long-term studies require a stable funding of the organizing institution, organized data holding and, in many cases, a close cooperation with educated volunteers (‘citizen scientists' [101]). Projects that aim to build up long-term studies on threatened species in the areas that have had little attention clearly deserve full support by decision-makers and project reviewers.

## 5. Conclusion

We found evidence for impacts of climatic factors on more than half of the species investigated. Changes in aspects of climate were found to have detrimental effects on one out of two amphibians and reptiles. Based on these results, climate change is already having and is anticipated to have a large impact on amphibian and reptilian species. Immediate and ambitious reductions in greenhouse gases [102] are of utmost importance for amphibians and reptiles of the world. Additionally, intensive general conservation efforts need to be employed to minimize today's main stressors on amphibians and reptiles, i.e. habitat loss [103] and overharvest, because these most likely will be aggravated by future climate changes. However, it is impossible to identify the most critical species to target for management given the lack of knowledge for some taxa and regions that we identified. Ideally, future research into the effects of climate change needs to focus on those regions, taxa and questions that to date have had little attention.

## Supplementary Material

Table S1. Search strings used to find literature for our meta-analysis. Table S2. Variables extracted for meta-analysis Table S3. Citation list of the literature reviewed in our study Table S4. List of species investigated by our reviewed studies, including 196 amphibian and 118 reptilian species, including unidentified species (sp.). Table S5. Comparison of the number of species investigated by the reviewed studies (“Nr. spp. studied”) and the total number of known species in each region (“Spp. in region”). Table S6. Comparison of the total number of amphibian and reptilian species within families (Spp. Total) with the number of species per family investigated by the reviewed studies (Spp. Studied)

## Data accessibility

Our dataset is available at the Dryad Data Repository: http://dx.doi.org/10.5061/dryad.54k37.
