# Supplementary material for: Patterns and biases in climate change research on amphibians and reptiles: a systematic review
Source: R Soc Open Sci. 2016 Sep 7;3(9):160158. doi: 10.1098/rsos.160158 (PMC5043301; doi:10.1098/rsos.160158)
Supplement: Table S1. Search strings used to find literature for our meta-analysis. Table S2. Variables extracted for meta-analysis Table S3. Citation list of the literature reviewed in our study Table S4. List of species investigated by our reviewed studies, including 196 amphibian and 118 reptilian species, i [file rsos160158supp1.pdf]

Table S1. Search strings used to find literature for our meta-analysis.

All papers included in our meta-analysis had to be non-experimental studies that investigate the effect of climate on amphibians and reptiles between 2005 and April 2015.

1. All studies on climate change, temperature or severe weather:
TS=(("climate change") OR (“warming world”) OR ("global warming") OR (change NEAR/5 climate*) OR (warming NEAR/5 climate*) OR (temperature OR "temperature increase" OR (warming NEAR/5 climate)) OR evapotranspiration OR moisture OR hydroperiod OR (severe weather OR storm OR hurricane* OR cyclone* OR tornado OR drought OR rain* OR precipitation OR snow* OR flood* OR blizzard))

2. All studies on amphibians or reptiles:
TS= (reptil* OR lizard* OR crocodile OR *turtle OR *snake) OR (*frog* OR amphib* OR salamander OR *toad OR newt OR anura*) OR herptile.

3. All studies on changes in species traits:
TS=(((chang* OR shift*) NEAR/5 population) OR ((chang* OR shift*) NEAR/5 lambda) OR ((chang* OR shift*) NEAR/5 distribution) OR ((chang* OR shift*) NEAR/5 phenolog*) OR ((chang* OR shift*) NEAR/5 dispersal) OR ((chang* OR shift*) NEAR/5 migration) OR ((chang* OR shift*) NEAR/5 elevation*) OR ((chang* OR shift*) NEAR/5 breeding) OR ((chang* OR shift*) NEAR/5 nesting) OR ((chang* OR shift*) NEAR/5 spawning) OR ((chang* OR shift*) NEAR/5 laying) OR ((chang* OR shift*) NEAR/5 abundance) OR mismatch* OR ((chang* OR shift*) NEAR/5 immune*) OR ((chang* OR shift*) NEAR/5 genetic*) OR ((chang* OR shift*) NEAR/5 survival*) OR ((increase* OR decreas*) NEAR/5 population*) OR ((increase* OR decreas*) NEAR/5 number*) OR ((chang* OR shift*) NEAR/5 development*) OR ((chang* OR shift*) NEAR/5 recruitment*) OR ((chang* OR shift*) NEAR/5 morpholog*) OR ((chang* OR shift*) NEAR/5 body) OR ((chang* OR shift*) NEAR/5 asymmetr*) OR disease OR competition OR “calling date” OR “breeding date” OR “spawning date”)

4. All studies that look at climate change, temperature, reptiles or amphibians and changes in species’ traits:
#1 AND #2 AND #3

5. Experimental studies, studies on agriculture and pests and studies on vegetation:
TS=((experiment* or test*) OR (agriculture* OR fodder OR pest OR milk or farm*) OR (tree OR vegetation OR forest OR leaf*))

6. All non-experimental studies that look at climate change, temperature, reptiles or amphibians and changes in species’ traits:
#4 NOT #5

Table S2. Variables extracted for meta-analysis

This table lists the variables for which information was extracted from each of the reviewed articles. Note that many pieces of information are binary and are coded as such: 0 (not investigated by the study) or 1 (investigated by the study), or, in case of the results: 0 (no effect documented) or 1 (effect was documented). Missing information is coded as "999999"); cells with this value will be excluded from analysis. For analysis we reduced the set of variables by forming more comprehensive variable groups (indicated by bold italic headings)

Extracted Variable
**General Information**
Author (last name)
Journal (Journal name)
Year (Year of publication)
Type of study (Field study or Modelling)
Number of years studied
Taxonomy (Class, Family, Genus, Species)
Conservation status
**Continent** ***(***North-, central- and South-America***,*** Europe, Africa***,*** Asia***,*** Middle East, Oceania)
**Location**
Exact place known? 0 or 1
Altitude (in meter; only for exact places; otherwise 999999)
Latitude: not included for global studies; for large-scale studies: midpoint of total study area
Longitude: not included for global studies; for large-scale studies: midpoint of total study area
Country: Name
**Study design**
Looked for changes through time (0 or 1)
**Predictor variables**
Climatic variables
Temperature (0 or 1)
Temperature Extreme (0 or 1)
Temperature variability (0 or 1)
Precipitation (0 or 1)
AMO (0 or 1)
SST (0 or 1)
NAO (0 or 1)
Evapotranspiration (0 or 1)
Hydroperiod (0 or 1)
Drought (0 or 1)
General moisture (0 or 1)
Wind-Storm, including hurricanes and tornadoes (0 or 1)
Water temperature (0 or 1)
Flooding (0 or 1)
Human impact variables
Fragmentation (0 or 1)
Habitat destruction (0 or 1)
Toxins (0 or 1)
Distance to human structures (0 or 1)
Introduced species (0 or 1)
Harvesting (0 or 1)

Human footprint (0 or 1)

General environmental variable

Radiation (0 or 1)

Vegetation cover (0 or 1)

Litter depth (0 or 1)

Habitat availability (0 or 1)

Soil type (0 or 1)

Presence of disease? (0 or 1)

Fire (0 or 1)

Competition (0 or 1)

Prey availability (0 or 1)

Response variables

Population

Population size (0 or 1)

Occurence (0 or 1)

Distribution

Distribution (0 or 1)

Population survival

Survival (0 or 1)

Roadkills (0 or 1)

Threat risk

Extinction probability (0 or 1)

Phenology

Phenology (0 or 1)

Calling behavior (0 or 1)

Spawn date (0 or 1)

Morphology

Body size (0 or 1)

Morphology (0 or 1)

Morphotype (0 or 1)

Reproduction

Clutch site (0 or 1)

Recruitment of Juvenils (0 or 1)

Hybridization rate (0 or 1)

Sex ratio (0 or 1)

Juvenile dispersal (0 or 1)

Disease

Presence of disease (0 or 1)

Physiology

Species physiology (0 or 1)

Development time (0 or 1)

Body condition (incl. mass) (0 or 1)

Immunology

Immune function (0 or 1)

Genetics

Genetics (0 or 1)

General analysis

Looked at changes over time? (0 or 1)

Modelled future changes? (0 or 1)

Results

General results

Significant climate effect for at least one species (1 or 0)

Nonclimatic factor investigated (0 or 1)

Climate main factor? (0 or 1, 2 for unknown)

Main climatic variable
(temperature, precipitation,…)

Variable change during the study
(-1, 0, 1)

Effect of climate variable on species
(-1, 0, 1; 2 for unclear)

Other factors but climate change discussed but not investigated?
(0 or 1)

Specific results

Pop. Increase
(0 or 1; 999999 for not applicable)

Pop. Decrease
(0 or 1; 999999 for not applicable)

Change in timing
(0 or 1; 999999 for not applicable)

Earlier
(0 or 1; 999999 for not applicable)

Later
(0 or 1¸999999 for not applicable)

Change in distribution
(0 or 1; 999999 for not applicable)

Change in habitat suitability (0 or 1)

Increase in suitable habitat (0 or 1; 999999 for not applicable)

Change in body size (0 or 1)

Change in extinction probability (0 or 1)

Decrease in survival (0 or 1)

Range contraction (0 or 1)

Change in reproductive success
(0 or 1)

Change in sex ratio (0 or 1)

Table S3. Citation list of the literature reviewed in our study

Aragon P, Rodriguez MA, Olalla-Tarraga MA, Lobo JM (2010) Predicted impact of climate change on threatened terrestrial vertebrates in central Spain highlights differences between endotherms and ectotherms. *Animal Conservation*, **13**, 363-373.

Aragon P, Rodriguez MA, Olalla-Tarraga MA, Lobo JM (2010) Predicted impact of climate change on threatened terrestrial vertebrates in central Spain highlights differences between endotherms and ectotherms. *Animal Conservation*, **13**, 363-373.

Aragón P, Lobo JM, Olalla-Tárraga MÁ, Rodríguez MÁ (2010) The contribution of contemporary climate to ectothermic and endothermic vertebrate distributions in a glacial refuge. *Global Ecology and Biogeography*, **19**, 40–49.

Araújo MB, Thuiller W, Pearson RG (2006) Climate warming and the decline of amphibians and reptiles in Europe. *Journal of Biogeography*, **33**, 1712–1728.

Arnfield H, Grant R, Monk C, Uller T (2012) Factors influencing the timing of spring migration in common toads (Bufo bufo). *Journal of Zoology*, **288**, 112–118.

Ballesteros-Barrera C, MartÍnez-Meyer E, Gadsden H (2007) Effects of Land-Cover Transformation and Climate Change on the Distribution of Two Microendemic Lizards, Genus Uma, of Northern Mexico. *Journal of Herpetology*, **41**, 733–740.

Barrows CW (2006) Population dynamics of a threatened sand dune lizard. *Southwest Naturalist*, **51**, 514–523.

Barrows CW, Fisher M. (2014) Past, present and future distributions of a local assemblage of congeneric lizards in southern California. Biological Conservation 180:97–107.

Barrows CW (2011) Sensitivity to climate change for two reptiles at the Mojave-Sonoran Desert interface. *Journal of Arid Environments*, **75**, 629–635.

Barrows CW, Allen MF (2009) Conserving species in fragmented habitats: Population dynamics of the Flat-tailed Horned Lizard. *Southwestern Naturalist*, **54**, 307–316.

Beebee TJC (2011) Modelling factors affecting population trends in an endangered amphibian. *Journal of Zoology*, **284**, 97–104.

Bombi P, Capula M, D’Amen M, Luiselli L (2011a) Climate change threatens the survival of highly endangered Sardinian populations of the snake Hemorrhois hippocrepis. *Animal Biology*, **61**, 239–248.

Bombi P, Akani GC, Ebere N, Luiselli L (2011b) Potential effects of climate change on high- and low-abundance populations of the Gaboon viper (Bitis gabonica) and the nose-horned viper (B. nasicornis) in southern Nigeria. *Herpetological Journal*, **21**, 59–64.

Brodman ROB (2009) A 14-year study of amphibian populations and metacommunities. *Herpetological Conserva tion and Biology*, **4**, 106–119.

Capula M, Rugiero L, Capizzi D et al. (2014) Long-term, climate change-related shifts in monthly patterns of roadkilled Mediterranean snakes (Hierophis viridiflavus). *Herpetological Journal*, **24**, 97–102.

Carroll EA, Sparks TH, Collinson N, Beebee TJB (2008) Influence of temperature on the spatial distribution of first spawning dates of the common frog (*Rana temporaria*) in the UK. *Global Change Biology*, **15**, 467–473.

Caruso NM, Lips KR (2013) Truly enigmatic declines in terrestrial salamander populations in Great Smoky Mountains National Park. *Diversity and Distributions*, **19**, 38–48.

Caruso NM, Sears MW, Adams DC, Lips KR (2014) Widespread rapid reductions in body size of adult salamanders in response to climate change. *Global Change Biology*, **20**, 1751–1759.

Ceia-Hasse A, Sinervo B, Vicente L, Pereira HM (2014) Integrating ecophysiological models into species distribution projections of European reptile range shifts in response to climate change. *Ecography*, **37**, 679–688.

Chadwick EA, Slater FM, Ormerod SJ (2006) Inter- and intraspecific differences in climatically mediated phenological change in coexisting Triturus species. *Global Change Biology*, **12**, 1069–1078.

Chamaille-Jammes S, Massot M, Aragon P, Clobert J (2006) Global warming and positive fitness response in mountain populations of common lizards Lacerta vivipara. *Global Change Biology*, **12**, 392–402.

D'Amen M, Bombi P (2009) Global warming and biodiversity: Evidence of climate-linked amphibian declines in Italy. *Biological Conservation*, **142**, 3060–3067.

Daszak P, Scott DE, Kilpatrick AM, Faggioni C, Gibbons JW, Porter D (2005) Amphibian population declines at Savannah River site are linked to climate, not chytridiomycosis. *Ecology*, **86**, 3232–3237.

Dodd Jr. CK, Hyslop NL, Oli MK (2012) The Effects of Disturbance Events on Abundance and Sex Ratios of a Terrestrial Turtle, Terrapene bauri. *Chelonian Conservation and Biology*, **11**, 44–49.

Dolgener N, Freudenberger L, Schneeweiss N, Ibisch PL, Tiedemann R (2013) Projecting current and potential future distribution of the Fire-bellied toad Bombina bombina under climate change in north-eastern Germany. *Regional Environmental Change*, **14**, 1063–1072.

Donald DB, Aitken WT, Paquette C, Wulff SS (2011) Winter snowfall determines the occupancy of northern prairie wetlands by tadpoles of the Wood Frog (Lithobates sylvaticus). *Canadian Journal of Zoology*, **89**, 1063–1073.

Encarnacion-Luevano A, Rojas-Soto OR, Jesus Sigala-Rodriguez J, Encarnación-Luévano A, Sigala-Rodríguez JJ (2013) Activity Response to Climate Seasonality in Species with Fossorial Habits: A Niche Modeling Approach Using the Lowland Burrowing Treefrog (Smilisca fodiens). *PLOS ONE*, **8**, e78290.

Fernandez-Chacon A, Bertolero A, Amengual A, Tavecchia G, Homar V, Oro D (2011) Spatial heterogeneity in the effects of climate change on the population dynamics of a Mediterranean tortoise. *Global Change Biology*, **17**, 3075–3088.

Forero-Medina G, Joppa L, Pimm SL (2011) Constraints to Species' Elevational Range Shifts as Climate Changes. *Conservation Biology*, **25**, 163–171.

Fouquet A, Ficetola GF, Haigh A, Gemmell N (2010) Using ecological niche modelling to infer past, present and future environmental suitability for Leiopelma hochstetteri, an endangered New Zealand native frog. *Biological Conservation*, **143**, 1375–1384.

Freedman AH, Buermann W, Lebreton M, Chirio L, Smith TB (2009) Modeling the Effects of Anthropogenic Habitat Change on Savanna Snake Invasions into African Rainforest. *Conservation Biology*, **23**, 81–92.

Fuentes MMPB, Abbs D (2010) Effects of projected changes in tropical cyclone frequency on sea turtles. *Marine Ecology Progress Series*, **412**, 283–292.

Gadsden H, Ballesteros-Barrera C, de la Garza O, Castaneda G, la Pena C, Lemos-Espinal JA (2012) Effects of land-cover transformation and climate change on the distribution of two endemic lizards, Crotaphytus antiquus and Sceloporus cyanostictus, of northern Mexico. *JOURNAL OF ARID ENVIRONMENTS*, **83**, 1–9.

Girardello M, Griggio M, Whittingham MJ, Rushton SP (2010) Models of climate associations and distributions of amphibians in Italy. *Ecological Research*, **25**, 103–111.

Green DM, Middleton J (2013) Body size varies with abundance, not climate, in an amphibian population. *Ecography*, **36**, 947–955.

Greenberg DA, Green DM (2013) Effects of an invasive plant on population dynamics in toads. *Conservation biology*, **27**, 1049–57.

Griffiths RA, Sewell D, McCrea RS (2010) Dynamics of a declining amphibian metapopulation: Survival, dispersal and the impact of climate. *Biological Conservation*, **143**, 485–491.

Güizado-Rodríguez MA, Ballesteros-Barrera C, Casas-Andreu G, Barradas-Miranda VL, Téllez-Valdés O, Salgado-Ugarte IH (2012) The impact of global warming on the range distribution of different climatic groups of Aspidoscelis costata costata. *Zoological science*, **29**, 834–43.

Hartel T (2008) Weather conditions, breeding date and population fluctuation in Rana dalmatina from central Romania. *Herpetological Journal*, **18**, 40–44.

Hawkes L a., Broderick a. C, Godfrey MH, Godley BJ (2007) Investigating the potential impacts of climate change on a marine turtle population. *Global Change Biology*, **13**, 923–932.

irschfeld M, Roedel M-O (2011) Variable reproductive strategies of an African savanna frog, Phrynomantis microps (Amphibia, Anura, Microhylidae). *Journal of Tropical Ecology*, **27**, 601–609.

Hof C, Araújo MB, Jetz W, Rahbek C (2011) Additive threats from pathogens, climate and land-use change for global amphibian diversity. *Nature*, **480**, 516–9.

Hossack BR, Corn PS (2007) Responses of pond-breeding amphibians to wildfire: Short-term patterns in occupancy and colonization. *Ecological Applications*, **17**, 1403–1410.

Houniet DT, Thuiller W, Tolley K a (2009) Potential effects of predicted climate change on the endemic South African Dwarf Chameleons, Bradypodion. *African Journal of Herpetology*, **58**, 28–35.

Jergenson AM, Miller DAW, Neuman-Lee LA, Warner DA, Janzen FJ (2014) Swimming against the tide: resilience of a riverine turtle to recurrent extreme environmental events. *Biology Letters*, **10**.

Klaus SP, Lougheed SC (2013) Changes in breeding phenology of eastern Ontario frogs over four decades. *Ecology and evolution*, **3**, 835–45.

Kubisch E, Piantoni C, Williams J, Scolaro A, Navas C a., Ibargüengoytía NR (2012) Do Higher Temperatures Increase Growth in the Nocturnal Gecko Homonota darwini (Gekkota: Phyllodactylidae)? A Skeletochronological Assessment Analyzed at Temporal and Geographic Scales. *Journal of Herpetology*, **46**, 587–595.

Kusano T, Inoue M (2008) Long-Term Trends toward Earlier Breeding of Japanese Amphibians. *Journal of Herpetology*, **42**, 608–614.

Lara-Reséndiz RA, Gadsden H, Rosen PC, Sinervo B, Méndez-De la Cruz FR (2015) Thermoregulation of two sympatric species of horned lizards in the Chihuahuan Desert and their local extinction risk. *Journal of thermal biology*, **48**, 1–10.

Laurance WF (2008) Global warming and amphibian extinctions in eastern Australia. *Austral Ecology*, **33**, 1–9.

Lelievre H, Rivalan P, Delmas V, Ballouard J-M, Bonnet X, Blouin-Demers G, Lourdais O (2013) The thermoregulatory strategy of two sympatric colubrid snakes affects their demography. *Population Ecology*, **55**, 585–593.

Lips KR, Brem F, Brenes R et al. (2006) Emerging infectious disease and the loss of biodiversity in a Neotropical amphibian community. *PNAS*, **103**, 3165–3170.

Lowe WH (2012) Climate change is linked to long-term decline in a stream salamander. *Biological Conservation*, **145**, 48–53.

Loyola RD, Lemes P, Brum FT, Provete DB, Duarte LDS (2014) Clade-specific consequences of climate change to amphibians in Atlantic Forest protected areas. *Ecography*, **37**, 65–72.

Luz Marquez A, Real R, Olivero J, Estrada A (2011) Combining climate with other influential factors for modelling the impact of climate change on species distribution. *Climate Change*, **108**, 135–157.

Martinez-Freiria F, Argaz H, Fahd S, Brito JC (2013) Climate change is predicted to negatively influence Moroccan endemic reptile richness. Implications for conservation in protected areas. *Naturwissenschaften*, **100**, 877–889.

Mazaris AD, Kallimanis AS, Sgardelis SP, Pantis JD (2008) Do long-term changes in sea surface temperature at the breeding areas affect the breeding dates and reproduction performance of Mediterranean loggerhead turtles? Implications for climate change. *Journal of Experimental Marine Biology and Ecology*, **367**, 219–226.

McCaffery R, Solonen A, Crone E (2012) Frog population viability under present and future climate conditions: a Bayesian state-space approach. *Journal of Animal Ecology*, **81**, 978–985.

McCallum ML (2010) Future climate change spells catastrophe for Blanchard's cricket frog, Acris blanchardi (Amphibia: Anura: Hylidae). *Acta Herpetologica*, **5**, 119–130.

McGrath AL, Lorenzen K (2010) Management history and climate as key factors driving natterjack toad population trends in Britain. *Animal Conservation*, **13**, 483–494.

McMenamin SK, Hadly E a, Wright CK (2008) Climatic change and wetland desiccation cause amphibian decline in Yellowstone National Park. *Proceedings of the National Academy of Sciences of the United States of America*, **105**, 16988–93.

Menendez-Guerrero PA, Graham CH (2013) Evaluating multiple causes of amphibian declines of Ecuador using geographical quantitative analyses. *Ecography*, **36**, 756–769.

Mitchell NJ, Kearney MR, Nelson NJ, Porter WP (2008) Predicting the fate of a living fossil: how will global warming affect sex determination and hatching phenology in tuatara? *Proceedings of the Royal Society B - Biological Sciences*, **275**, 2185–2193.

Moreno-Rueda G, Pleguezuelos JM, Alaminos E (2009) Climate warming and activity period extension in the Mediterranean snake Malpolon monspessulanus. *Climatic Change*, **92**, 235–242.

Moreno-Rueda G, Pleguezuelos JM, Pizarro M, Montori A (2012) Northward Shifts of the Distributions of Spanish Reptiles in Association with Climate Change. *Conservation Biology*, **26**, 278–283.

Moskwik M (2014) Recent elevational range expansions in plethodontid salamanders (Amphibia: Plethodontidae) in the southern Appalachian Mountains. *Journal of Biogeography*, **41**, 1957–1966.

Muths E, Pilliod DS, Livo LJ (2008) Distribution and environmental limitations of an amphibian pathogen in the Rocky Mountains, USA. *Biological Conservation*, **141**, 1484–1492.

Narins PM, Meenderink SWF (2014) Climate change and frog calls: long-term correlations along a tropical altitudinal gradient. *Proceedings of the Royal Society B - Biological Sciences*, **281**.

Neveu A (2009) Incidence of climate on common frog breeding: Long-term and short-term changes. *Acta Oecologica*, **35**, 671–678.

Nori J, Urbina-Cardona JN, Loyola RD, Lescano JN, Leynaud GC (2011) Climate Change and American Bullfrog Invasion: What Could We Expect in South America? PLoS ONE. 6(10,

Nori J, Carrasco PA, Leynaud GC (2014) Venomous snakes and climate change: ophidism as a dynamic problem. *Climatic Change*, **122**, 67–80.

Olsson M, Schwartz T, Wapstra E, Uller T, Ujvari B, Madsen T, Shine R (2011) Climate change, multiple paternizy and offspring survival in lizards. *Evolution*, **65**, 3323–3326.

Parra-Olea G, Martinez-Meyer E, de Leon GFP (2005) Forecasting climate change effects on salamander distribution in the highlands of central Mexico. *Biotropica*, **37**, 202–208.

Pellet J, Schmidt BR, Fivaz F, Perrin N, Grossenbacher K (2006) Density, climate and varying return points: An analysis of long-term population fluctuations in the threatened European tree frog. *Oecologia*, **149**, 65–71.

Penman TD, Pike DA, Webb JK, Shine R (2010) Predicting the impact of climate change on Australia's most endangered snake, Hoplocephalus bungaroides. *Diversity and Distribution*, **16**, 109–118.

Phillimore AB, Hadfield JD, Jones OR, Smithers RJ (2010) Differences in spawning date between populations of common frog reveal local adaptation. *Proceedings of the National Academy of Sciences of the United States of America*, **107**, 8292–7.

Pike D a. (2014) Forecasting the viability of sea turtle eggs in a warming world. *Global Change Biology*, **20**, 7–15.

Pike DA, Antworth RL, Stiner JC (2006) Earlier nesting contributes to shorter nesting seasons for the Loggerhead Seaturtle, Caretta caretta. *Journal of Herpetology*, **40**, 91–94.

Pomara LY, Ledee OE, Martin KJ, Zuckerberg B (2014) Demographic consequences of climate change and land cover help explain a history of extirpations and range contraction in a declining snake species. *Global Change Biology*, **20**, 2087–2099.

Popescu VD, Rozylowicz L, Cogalniceanu D, Niculae IM, Cucu AL (2013) Moving into Protected Areas? Setting Conservation Priorities for Romanian Reptiles and Amphibians at Risk from Climate Change. *PLOS ONE*, **8**.

Pounds JA, Bustamante MR, Coloma L a et al. (2006) Widespread amphibian extinctions from epidemic disease driven by global warming. *Nature*, **439**, 161–7.

Primack RB, Ibanez I, Higuchi H, Lee SD, Miller-Rushing AJ, Wilson AM, Silander Jr. JA (2009) Spatial and interspecific variability in phenological responses to warming temperatures. *Biological Conservation*, **142**, 2569–2577.

Reading CJ (2007) Linking global warming to amphibian declines through its effects on female body condition and survivorship. *Oecologia*, **151**, 125–131.

Reece JS, Passeri D, Ehrhart L et al. (2013) Sea level rise, land use, and climate change influence the distribution of loggerhead turtle nests at the largest USA rookery (Melbourne Beach, Florida). *Marine Ecology Progress Series*, **493**, 259–274.

Roedder D, Loetters S (2010) Explanative power of variables used in species distribution modelling: an issue of general model transferability or niche shift in the invasive Greenhouse frog (Eleutherodactylus planirostris). *Naturwissenschaften*, **97**, 781–796.

Rohr JR, Raffel TR (2010) Linking global climate and temperature variability to widespread amphibian declines putatively caused by disease. *PNAS*, **107**, 8269–8274.

Rugiero L, Milana G, Petrozzi F, Capula M, Luiselli L (2013) Climate-change-related shifts in annual phenology of a temperate snake during the last 20 years. *Acta Oecologica*, **51**, 42–48.

Ryan MJ, Fuller MM, Scott NJ et al. (2014) Individualistic Population Responses of Five Frog Species in Two Changing Tropical Environments over Time. *PLOS ONE*, **9**.

Sahlean TC, Gherghel I, Papes M, Strugariu A, Zamfirescu SR (2014) Refining Climate Change Projections for Organisms with Low Dispersal Abilities: A Case Study of the Caspian Whip Snake. *PLOS ONE*, **9**.

Scherer RD, Muths E, Lambert BA (2008) Effects of weather on survival in populations of boreal toads in Colorado. *Journal of Herpetology*, **42**, 508–517.

Schwanz LE, Janzen FJ (2015) Climate change and temperature-dependent sex determination: can individual plasticity in nesting phenology prevent extreme sex ratios? *Physiological and biochemical zoology : PBZ*, **81**, 826–834.

Scott WA, Pithart D, Adamson JK (2008) Long-term United Kingdom trends in the breeding phenology of the Common Frog, Rana temporaria. *Journal of Herpetology*, **42**, 89–96.

Sillero N, Carretero M a. (2013) Modelling the past and future distribution of contracting species. The Iberian lizard Podarcis carbonelli (Squamata: Lacertidae) as a case study. *Zoologischer Anzeiger*, **252**, 289–298.

Sinervo B, Méndez-de-la-Cruz F, Miles DB et al. (2010) Erosion of lizard diversity by climate change and altered thermal niches. *Science (New York, N.Y.)*, **328**, 894–988.

Sodhi NS, Bickford D, Diesmos AC et al. (2008) Measuring the meltdown: Drivers of global amphibian extinction and decline. *PLoS ONE*, **3**, 1–8.

Sparks T, Tryjanowski P, Cooke A, Crick H, Kuzniak S. Vertebrate phenology at similar latitudes: temperature responses differ between Poland and the United Kingdom. Clim Res. 2007 Jul;34(2):93–8.

Telemeco RS, Elphick MJ, Shine R (2009) Nesting lizards (Bassiana duperreyi) compensate partly, but not completely, for climate change. *Ecology*, **90**, 17–22.

Tingley R, Herman TB (2009) Land-cover data improve bioclimatic models for anurans and turtles at a regional scale. *Journal of Biogeography*, **36**, 1656–1672.

Todd BD, Scott DE, Pechmann JHK, Gibbons JW (2011) Climate change correlates with rapid delays and advancements in reproductive timing in an amphibian community. *Proceedings of the Royal Society B - Biological Sciences*, **278**, 2191–2197.

Tryjanowski P, Sparks T, Rybacki M, Berger L (2006) Is body size of the water frog Rana esculenta complex responding to climate change? *Die Naturwissenschaften*, **93**, 110–3.

Walker SF, Bosch J, Gomez V et al. (2010) Factors driving pathogenicity vs. prevalence of amphibian panzootic chytridiomycosis in Iberia. *Ecology Letters*, **13**, 372–382.

Walls SC (2009) The role of climate in the dynamics of a hybrid zone in Appalachian salamanders. *Global Change Biology*, **15**, 1903–1910.

Wapstra E, Uller T, Sinn DL, Olsson M, Mazurek K, Joss J, Shine R (2009) Climate effects on offspring sex ratio in a viviparous lizard. *Journal of Animal Ecology*, **78**, 84–90.

Westervelt JD, Sperry JH, Burton JL, Palis JG (2013) Modeling response of frosted flatwoods salamander populations to historic and predicted climate variables. *Ecological Modeling*, **268**, 18–24.

Whitfield SM, Bell KE, Philippi T et al. (2007) Amphibian and reptile declines over 35 years at La Selva, Costa Rica. *Proceedings of the National Academy of Sciences of the United States of America*, **104**, 8352–6.

Zank C, Becker FG, Abadie M, Baldo D, Maneyro R, Borges-Martins M (2014) Climate Change and the Distribution of Neotropical RedBellied Toads (Melanophryniscus, Anura, Amphibia): How to Prioritize Species and Populations? *PLOS ONE*, **9**.

Zylstra ER, Steidl RJ, Jones CA, Averill-Murray RC (2013) Spatial and temporal variation in survival of a rare reptile: a 22-year study of Sonoran desert tortoises. *Oecologia*, **173**, 107–116.

Table S4. List of species investigated by our reviewed studies, including 196 amphibian and 118 reptilian species, including unidentified species (sp.).

Amphibia			Reptilia		
**Family**	**Genus**	**Species**	**Family**	**Genus**	**Species**
Alytidae	Alytes	dickhilleni	Anguidae	Anguis	fragilis
Alytidae	Alytes	obstetricans	Blanidae	Blanus	cinereus
Alytidae	Discoglossus	pictus	Blanidae	Blanus	mettetali
Alytidae	Discoglossus	sardus	Blanidae	Blanus	tingitanus
Ambystomatidae	Ambystoma	cingulatum	Chamaeleonidae	Bradypodion	damaranum
Ambystomatidae	Ambystoma	laterale	Chamaeleonidae	Bradypodion	gutturale
Ambystomatidae	Ambystoma	macrodactylum	Chamaeleonidae	Bradypodion	melanocephalum
Ambystomatidae	Ambystoma	opacum	Chamaeleonidae	Bradypodion	occidentale
Ambystomatidae	Ambystoma	talpoideum	Chamaeleonidae	Bradypodion	pumilum
Ambystomatidae	Ambystoma	tigrinum	Chamaeleonidae	Bradypodion	taeniabronchum
Bombinatoridae	Bombina	bombina	Chamaeleonidae	Bradypodion	transvaalense
Bombinatoridae	Bombina	pachypus	Chamaeleonidae	Bradypodion	ventrale
Bombinatoridae	Bombina	variegata	Chamaeleonidae	Chamaeleo	chamaeleon
Bufonidae	Anaxyrus	fowleri	Cheloniidae	Caretta	caretta
Bufonidae	Atelopus	arsyecue	Cheloniidae	Chelonia	mydas
Bufonidae	Atelopus	carrikeri	Cheloniidae	Eretmochelys	imbricata
Bufonidae	Atelopus	laetissimus	Cheloniidae	Natator	depresus
Bufonidae	Atelopus	nahumae	Colubridae	Coluber	caspius
Bufonidae	Atelopus	walkeri	Colubridae	Coronella	austriaca
Bufonidae	Bufo	americanus	Colubridae	Coronella	girondica
Bufonidae	Bufo	boreas	Colubridae	Dolichophis	caspius
Bufonidae	Bufo	bufo	Colubridae	Elaphe	longissima
Bufonidae	Bufo	bufo	Colubridae	Elaphe	quatuorlineata
Bufonidae	Bufo	calamita	Colubridae	Hemorrhois	hippocrepis
Bufonidae	Bufo	fowleri	Colubridae	Hierophis	viridiflavus
Bufonidae	Bufo	quercicus	Colubridae	Macroprotodon	brevis
Bufonidae	Bufo	sp.	Colubridae	Rhinechis	scalaris
Bufonidae	Bufo	terrestris	Colubridae	Zamenis	longissimus
Bufonidae	Bufo	viridis	Crotaphytidae	Crotaphytus	antiquus
Bufonidae	Melanophryniscus	atroluteus	Elapidae	Hoplocephalus	bungaroides
Bufonidae	Melanophryniscus	cambaraensis	Elapidae	Micrurus	pyrrhocryptus
Bufonidae	Melanophryniscus	cupreuscapularis	Emydidae	Chrysemys	picta
Bufonidae	Melanophryniscus	devincenzii	Emydidae	Emys	orbiculari
Bufonidae	Melanophryniscus	dorsalis	Emydidae	Emys	orbicularis
Bufonidae	Melanophryniscus	estebani	Emydidae	Terrapene	bauri
Bufonidae	Melanophryniscus	fulvoguttatus	Emydidae	Terrapene	carolina
Bufonidae	Melanophryniscus	klappenbachi	Gekkonidae	Hemidactylus	turcicus
Bufonidae	Melanophryniscus	krauczuki	Geoemydidae	Mauremys	leprosa
Bufonidae	Melanophryniscus	langonei	Iguanidae	Sauromalus	ater

Amphibia			Reptilia		
**Family**	**Genus**	**Species**	**Family**	**Genus**	**Species**
Bufonidae	Melanophryniscus	macrogranulosus	Lacertidae	Acanthodactylus	busacki
Bufonidae	Melanophryniscus	montevidensis	Lacertidae	Acanthodactylus	erythrurus
Bufonidae	Melanophryniscus	moreirae	Lacertidae	Acanthodactylus	lineomaculatus
Bufonidae	Melanophryniscus	pachyrhynus	Lacertidae	Atlantolacerta	andreanskyi
Bufonidae	Melanophryniscus	paraguayensis	Lacertidae	Eremias	arguta
Bufonidae	Melanophryniscus	rubriventris	Lacertidae	Iberolacerta	monticola
Bufonidae	Melanophryniscus	sanmartini	Lacertidae	Lacerta	agilis
Bufonidae	Melanophryniscus	simplex	Lacertidae	Lacerta	bilineata
Bufonidae	Melanophryniscus	spectabilis	Lacertidae	Lacerta	lepida
Bufonidae	Melanophryniscus	sp1	Lacertidae	Lacerta	praticola
Bufonidae	Melanophryniscus	sp2	Lacertidae	Lacerta	schreiberi
Bufonidae	Melanophryniscus	sp3	Lacertidae	Lacerta	trilineata
Bufonidae	Melanophryniscus	stelzneri	Lacertidae	Lacerta	viridis
Bufonidae	Melanophryniscus	tumifrons	Lacertidae	Lacerta	vivipara
Bufonidae	Rhaebo	haematiticus	Lacertidae	Podarcis	carbonelli
Bufonidae	Rhinella	beebei	Lacertidae	Podarcis	hispanica
Bufonidae	Rhinella	granulosa	Lacertidae	Podarcis	muralis
Bufonidae	Rhinella	marina	Lacertidae	Podarcis	taurica
Caeciliidae	Caecilia	subnigricans	Lacertidae	Psammodromus	algirus
Centrolenidae	Centrolene	tayrona	Lacertidae	Psammodromus	hispanicus
Ceratophryidae	Ceratophrys	calcarata	Lacertidae	Timon	lepidus
Craugastoridae	Craugastor	bransfordii	Lacertidae	Timon	tangitanus
Craugastoridae	Craugastor	crassidigitus	Lacertidae	Zootoca	vivipara
Craugastoridae	Craugastor	fitzingeri	Lamprophiidae	Lamprophis	fuliginosus
Craugastoridae	Craugastor	megacephalus	Lamprophiidae	Malpolon	monspessulanus
Craugastoridae	Craugastor	mimus	Lamprophiidae	Psammophis	lineatus
Craugastoridae	Craugastor	noblei	Natricidae	Natrix	maura
Craugastoridae	Craugastor	rugosus	Natricidae	Natrix	natrix
Craugastoridae	Craugastor	stejnegerianus	Natricidae	Natrix	tessellata
Craugastoridae	Craugastor	talamancae	Phrynosomatidae	Phrynosoma	cornutum
Dendrobatidae	Colostethus	ruthveni	Phrynosomatidae	Phrynosoma	mcallii
Dendrobatidae	Oophaga	pumilio	Phrynosomatidae	Phrynosoma	modestum
Eleutherodactylidae	Diasporus	vocator	Phrynosomatidae	Sceloporus	cyanostictus
Eleutherodactylidae	Eleutherodactylus	caryophyllaceus	Phrynosomatidae	Sceloporus	magister
Eleutherodactylidae	Eleutherodactylus	cerasinus	Phrynosomatidae	Sceloporus	occidentalis
Eleutherodactylidae	Eleutherodactylus	coqui	Phrynosomatidae	Sceloporus	orcutti
Eleutherodactylidae	Eleutherodactylus	cruentus	Phrynosomatidae	Sceloporus	vandenburgianus
Eleutherodactylidae	Eleutherodactylus	diastema	Phrynosomatidae	Uma	exsul
Eleutherodactylidae	Eleutherodactylus	ridens	Phrynosomatidae	Uma	inornata
Hemiphractidae	Cryptobatrachus	boulengeri	Phrynosomatidae	Uma	paraphygas
Hylidae	Acris	blanchardi	Phyllodactylidae	Homonota	darwini
Hylidae	Acris	crepitans	Phyllodactylidae	Tarentola	boehmei
Hylidae	Acris	gryllus	Phyllodactylidae	Tarentola	mauritanica

Amphibia			Reptilia		
**Family**	**Genus**	**Species**	**Family**	**Genus**	**Species**
Hylidae	Dendropsophus	microcephalus	Scincidae	Ablepharus	kitaibelii
Hylidae	Hyla	arborea	Scincidae	Bassiana	duperreyi
Hylidae	Hyla	chrysoscelis	Scincidae	Chalcides	colosii
Hylidae	Hyla	cinerea	Scincidae	Chalcides	lanzai
Hylidae	Hyla	gratiosa	Scincidae	Chalcides	minutus
Hylidae	Hyla	intermedia	Scincidae	Chalcides	mionecton
Hylidae	Hyla	sarda	Scincidae	Chalcides	montanus
Hylidae	Hyla	versicolor	Scincidae	Chalcides	polylepis
Hylidae	Hypsiboas	crepitans	Scincidae	Chalcides	pseudostriatus
Hylidae	Hypsiboas	pugnax	Scincidae	Chalcides	striatus
Hylidae	Phyllomedusa	venusta	Scincidae	Niveoscincus	ocellatus
Hylidae	Pseudacris	crucifer	Sphaerodactylidae	Quedenfeldtia	moerens
Hylidae	Pseudacris	ornata	Sphaerodactylidae	Saurodactylus	brosseti
Hylidae	Pseudacris	spp	Sphaerodactylidae	Saurodactylus	fasciatus
Hylidae	Pseudacris	triseriata	Sphenodontidae	Sphenodon	guntheri
Hylidae	Pseudis	paradoxa	Teiidae	Aspidoscelis	costata
Hylidae	Scinax	rostratus	Testudinidae	Gopherus	agassizii
Hylidae	Scinax	ruber	Testudinidae	Gopherus	morafkai
Hylidae	Scinax	x-signatus	Testudinidae	Testudo	graeca
Hylidae	Smilisca	fodiens	Testudinidae	Testudo	hermanni
Hylidae	Trachycephalus	venulosus	Viperidae	Bitis	gabonica
Hynobiidae	Hynobius	tokyoensis	Viperidae	Bitis	nasicornis
Leiopelmatidae	Leiopelma	hochstetteri	Viperidae	Bothrops	alternatus
Leptodactylidae	Engystomops	pustulosus	Viperidae	Bothrops	ammodytoides
Leptodactylidae	Leptodactylus	bolivianus	Viperidae	Bothrops	diporus
Leptodactylidae	Leptodactylus	fuscus	Viperidae	Causus	maculatus
Leptodactylidae	Leptodactylus	pentadactylus	Viperidae	Crotalus	durissus
Leptodactylidae	Leptodactylus	poecilochilus	Viperidae	Sistrurus	catenatus
Leptodactylidae	Pleurodema	brachyops	Viperidae	Vipera	ammodytes
Leptodactylidae	Pseudopaludicola	pusilla	Viperidae	Vipera	aspis
Microhylidae	Chiasmocleis	panamensis	Viperidae	Vipera	berus
Microhylidae	Elachistocleis	ovalis	Viperidae	Vipera	latastei
Microhylidae	Elachistocleis	pearsei	Viperidae	Vipera	monticola
Microhylidae	Gastrophryne	carolinensis	Viperidae	Vipera	seoanei
Microhylidae	Gastrophryne	pictiventris	Viperidae	Vipera	ursinii
Microhylidae	Phrynomantis	microps			
Pelobatidae	Pelobates	fuscus			
Pelobatidae	Pelobates	syriacus			
Plethodontidae	Bolitoglossa	savagei			
Plethodontidae	Desmognathus	organi			
Plethodontidae	Eurycea	quadridigitata			
Plethodontidae	Gyrinophilus	porphyriticus			
Plethodontidae	Plethodon	aureolus			

Amphibia			Reptilia		
Family	Genus	Species	Family	Genus	Species
Plethodontidae	Plethodon	cheoah			
Plethodontidae	Plethodon	cinereus			
Plethodontidae	Plethodon	cylindraceus			
Plethodontidae	Plethodon	glutinosus			
Plethodontidae	Plethodon	jordani			
Plethodontidae	Plethodon	metcalfi			
Plethodontidae	Plethodon	montanus			
Plethodontidae	Plethodon	nettingi			
Plethodontidae	Plethodon	richmondi			
Plethodontidae	Plethodon	serratus			
Plethodontidae	Plethodon	shermani			
Plethodontidae	Plethodon	sp.			
Plethodontidae	Plethodon	teyahaleea			
Plethodontidae	Plethodon	ventralis			
Plethodontidae	Plethodon	welleri			
Plethodontidae	Plethodon	yonahlosseea			
Plethodontidae	Pseudoeurycea	cephalica			
Plethodontidae	Pseudoeurycea	leprosa			
Ranidae	Pelophylax	esculenta			
Ranidae	Pelophylax	lessonae			
Ranidae	Pelophylax	nigromaculata			
Ranidae	Pelophylax	sp.			
Ranidae	Rana	arvalis			
Ranidae	Rana	catesbeianus			
Ranidae	Rana	clamitans			
Ranidae	Rana	dalmatina			
Ranidae	Rana	grylio			
Ranidae	Rana	italica			
Ranidae	Rana	latastei			
Ranidae	Rana	luteiventris			
Ranidae	Rana	ornativentris			
Ranidae	Rana	palustris			
Ranidae	Rana	pipiens			
Ranidae	Rana	porosa			
Ranidae	Rana	ridibunda			
Ranidae	Rana	septentrionalis			
Ranidae	Rana	sphenocephala			
Ranidae	Rana	sylvatica			
Ranidae	Rana	temporaria			
Ranidae	Rana	vaillanti			
Ranidae	Rana	virgatipes			
Ranidae	Rana	warszewitschii			
Rhacophoridae	Rhacophorus	arboreus			

Amphibia			Reptilia		
**Family**	**Genus**	**Species**	**Family**	**Genus**	**Species**
Salamandridae	Ichthyosaura	alpestris			
Salamandridae	Lissotriton	helveticus			
Salamandridae	Lissotriton	italicus			
Salamandridae	Lissotriton	montandoni			
Salamandridae	Lissotriton	vulgaris			
Salamandridae	Notophthalmus	viridescens			
Salamandridae	Salamandra	atra			
Salamandridae	Salamandra	salamandra			
Salamandridae	Salamandrina	sp.			
Salamandridae	Salamandrina	tergiditata			
Salamandridae	Triturus	carnifex			
Salamandridae	Triturus	cristatus			
Salamandridae	Triturus	dobrogicus			
Scaphiopodidae	Scaphiopus	holbrookii			
Sirenidae	Siren	intermedia			
Strabomantidae	Geobatrachus	walkeri			
Strabomantidae	Pristimantis	carmelitae			
Strabomantidae	Pristimantis	cristinae			
Strabomantidae	Pristimantis	delicatus			

Table S5. Comparison of the number of species investigated by the reviewed studies ("Nr. spp. studied") and the total number of known species in each region ("Spp. in region"), indicating a great bias of studying species on continents with low species diversity. "% spp studied" shows the percentage of the total number of species in the region that was investigated by the reviewed studies.

	Amphibia			Reptilia		
**Region**	Nr. spp. studied	Spp. in region	% spp. studied	Nr. spp. studied	Spp. in region	% spp. studied
Europe	38	90	42	55	165	33
North America	60	279	21	21	1009	2
Central America	22	731	3	0	864	0
South America	71	2377	3	6	1982	0.3
Carribean	1	218	0.5	0	500	0
Asia	5	1519	0.3	4	2976	0.1
Australia + Oceania	1	494	0.2	4	1267	0.3
Africa	1	1122	0.1	31	2085	1.5

[1] We used the number of species assessments by the IUCN as an approximation for the total number of amphibian species on each continent. This number is lower than the actual number of species.

[2]The number of reptiles were summarized from data from the reptilian database by Yuval Itescu and Anat Feldman. Some species occur in several geographic regions. Therefore, the total number of reptilian species adds up to more than the total number of known reptilian species.

Table S6. Comparison of the total number of amphibian and reptilian species within families (Spp. Total) with the number of species per family investigated by the reviewed studies (Spp. Studied); also listed is the percentage of species studied per family, compared to the total number of species (%).

Amphibians				Reptiles			
Family	Spp. Total	Spp. Studied	%	Family	Total Spp.	Nr. spp. studied	%
Allophrynidae	3	0	0,00	Acrochordidae	3	0	0
Alsodidae	30	0	0,00	Agamidae	456	0	0,00
Alytidae	11	4	36,36	Alligatoridae	8	0	0,00
Ambystomatidae	32	6	18,75	Amphisbaenidae	173	0	0,00
Amphiumidae	3	0	0,00	Anguidae	73	1	1,37
Arthroleptidae	149	0	0,00	Aniliidae	1	0	0,00
Ascaphidae	2	0	0,00	Anniellidae	6	0	0,00
Batrachylidae	15	0	0,00	Anomalepididae	18	0	0,00
Bombinatoridae	10	3	30,00	Anomochilidae	3	0	0,00
Brachycephalidae	54	0	0,00	Bipedidae	3	0	0,00
Brevicipitidae	34	0	0,00	Blanidae	7	3	42,86
Bufonidae	592	45	7,60	Boidae	58	0	0,00
Caeciliidae	42	1	2,38	Bolyeridae	2	0	0,00
Calyptocephalellidae	4	0	0,00	Cadeidae	2	0	0,00
Centrolenidae	153	1	0,65	Carettochelyidae	1	0	0,00
Ceratobatrachidae	87	0	0,00	Carphodactylidae	30	0	0,00
Ceratophryidae	12	1	8,33	Chamaeleonidae	202	9	4,46
Ceuthomantidae	4	0	0,00	Chelidae	56	0	0,00
Chikilidae	4	0	0,00	Cheloniidae	6	4	66,67
Conrauidae	6	0	0,00	Chelydridae	6	0	0,00
Craugastoridae	116	9	7,76	Colubridae	844	11	1,30
Cryptobranchidae	3	0	0,00	Cordylidae	66	0	0,00
Cycloramphidae	34	0	0	Corytophanidae	9	0	0,00
Dendrobatidae	303	2	0,66	Crocodylidae	16	0	0,00
Dermophiidae	14	0	0,00	Crotaphytidae	12	1	8,33
Dicamptodontidae	4	0	0,00	Cylindrophiidae	10	0	0,00
Dicroglossidae	195	0	0,00	Dactyloidae	398	0	0,00
Eleutherodactylidae	210	7	3,33	Dermatemydidae	1	0	0,00
Heleophrynidae	6	0	0,00	Dermochelyidae	1	0	0,00
Hemiphractidae	103	1	0,97	Dibamidae	23	0	0,00
Hemisotidae	9	0	0,00	Diplodactylidae	130	0	0,00
Herpelidae	9	0	0,00	Diploglossidae	51	0	0,00

Amphibians				Reptiles			
Family	Spp. Total	Spp. Studied	%	Family	Total Spp.	Nr. spp. studied	%
Hylidae	950	24	2,53	Dipsadidae	752	0	0,00
Hylodidae	46	0	0,00	Elapidae	355	2	0,56
Hynobiidae	64	1	1,56	Emydidae	52	5	9,62
Hyperoliidae	223	0	0,00	Eublepharidae	34	0	0,00
Ichthyophiidae	58	0	0,00	Gavialidae	1	0	0,00
Indotyphlidae	21	0	0,00	Gekkonidae	1033	1	0,10
Leiopelmatidae	4	1	25,00	Geoemydidae	69	1	1,45
Leptodactylidae	205	7	3,41	Gerrhopilidae	18	0	0,00
Mantellidae	207	0	0,00	Gerrhosauridae	37	0	0,00
Megophryidae	190	0	0,00	Gymnophthalmidae	246	0	0,00
Micrixalidae	24	0	0,00	Helodermatidae	2	0	0,00
Microhylidae	572	6	1,05	Homalopsidae	53	0	0,00
Myobatrachidae	133	0	0,00	Hoplocercidae	16	0	0,00
Nasikabatrachidae	1	0	0,00	Iguanidae	41	1	2,44
Nyctibatrachidae	29	0	0,00	Kinosternidae	25	0	0,00
Odontobatrachidae	1	0	0,00	Lacertidae	321	23	7,17
Odontophrynidae	52	0	0,00	Lamprophiidae	309	3	0,97
Pelobatidae	4	2	50,00	Lanthanotidae	1	0	0,00
Pelodytidae	3	0	0,00	Leiocephalidae	29	0	0,00
Petropedetidae	12	0	0,00	Leiosauridae	33	0	0,00
Phrynobatrachidae	88	0	0,00	Leptotyphlopidae	119	0	0,00
Pipidae	33	0	0,00	Liolaemidae	288	0	0,00
Plethodontidae	446	23	5,16	Loxocemidae	1	0	0,00
Proteidae	6	0	0,00	Natricidae	226	3	1,33
Ptychadenidae	52	0	0,00	Opluridae	7	0	0,00
Pyxicephalidae	80	0	0,00	Pareatidae	18	0	0,00
Ranidae	379	25	6,60	Pelomedusidae	27	0	0,00
Ranixalidae	11	0	0,00	Phrynosomatidae	148	11	7,43
Rhacophoridae	391	1	0,26	Phyllodactylidae	134	3	2,24
Rhinatrematidae	11	0	0,00	Platysternidae	1	0	0,00
Rhinodermatidae	3	0	0,00	Podocnemididae	8	0	0,00
Rhinophrynidae	1	0	0,00	Polychrotidae	7	0	0,00
Rhyacotritonidae	4	0	0,00	Pseudoxenodontidae	11	0	0,00
Salamandridae	110	13	11,82	Pygopodidae	44	0	0,00
Scaphiopodidae	7	1	14,29	Pythonidae	40	0	0,00
Scolecomorphidae	6	0	0,00	Rhineuridae	1	0	0,00
Siphonopidae	26	0	0,00	Scincidae	1589	11	0,69

Amphibians				Reptiles			
**Family**	**Spp. Total**	**Spp. Studied**	**%**	**Family**	**Total Spp.**	**Nr. spp. studied**	**%**
Sirenidae	4	1	25,00	Shinisauridae	1	0	0,00
Sooglossidae	4	0	0,00	Sphaerodactylidae	213	3	1,41
Strabomantidae	623	11	1,77	Sphenodontidae	1	1	100,00
Telmatobiidae	63	0	0,00	Teiidae	150	1	0,67
Typhlonectidae	13	1	7,69	Testudinidae	57	4	7,02
				Trionychidae	31	0	0,00
				Trogonophiidae	6	0	0,00
				Tropidophiidae	34	0	0,00
				Tropiduridae	125	0	0,00
				Typhlopidae	261	0	0,00
				Uropeltidae	54	0	0,00
				Varanidae	78	0	0,00
				Viperidae	329	15	4,56
				Xantusiidae	34	0	0,00
				Xenodermatidae	18	0	0,00
				Xenopeltidae	2	0	0,00
				Xenophidiidae	2	0	0,00
				Xenosauridae	10	0	0,00
				Xenotyphlopidae	1	0	0,00
